# Nonsuicidal Self-Injury: Its Associations With Pathological Internet Use and Psychopathology Among Adolescents

**DOI:** 10.3389/fpsyt.2020.00814

**Published:** 2020-08-14

**Authors:** Gergely Mészáros, Dora Győri, Lili Olga Horváth, Dora Szentiványi, Judit Balázs

**Affiliations:** ^1^ Department of Psychiatry and Psychotherapy, Faculty of Medicine, Semmelweis University, Budapest, Hungary; ^2^ Mental Health Sciences School of Ph.D., Semmelweis University, Budapest, Hungary; ^3^ Doctoral School of Psychology, Eötvös Loránd University, Budapest, Hungary; ^4^ Institute of Psychology, Eötvös Loránd University, Budapest, Hungary; ^5^ Pedagogical Assistance Services, Budapest, Hungary; ^6^ Department of Psychology, Bjørknes University College, Oslo, Norway

**Keywords:** NSSI: nonsuicidal self-injury, PIU: pathological internet use, internet addiction, internalization, externalization, psychopathology, adolescent

## Abstract

**Background/Hypotheses:**

As risk factors for nonsuicidal self-injury (NSSI), most studies highlight the importance of internalising disorders, while only a few researches show the connection between externalising disorders and NSSI. Although some papers have introduced the idea that increasing prevalence rates of NSSI are connected to the broader use of the internet, associations between NSSI and pathological internet use (PIU) are understudied. According to our hypothesis, there is a connection between PIU and NSSI, but this is mediated by psychopathological factors from both internalising and externalising dimensions.

**Methods:**

In line with the dimensional approach of psychiatric disorders, participants (N = 363) were recruited from both clinical (N = 202 psychiatric inpatient) and nonclinical (N = 161 adolescents from secondary schools) settings. Measurements: Demographic Questionnaire; Strengths and Difficulties Questionnaire (SDQ); Deliberate Self-Harm Inventory (DSHI); Young Diagnostic Questionnaire for Internet Addiction (YDQ), Mini International Neuropsychiatric Interview Kid (M.I.N.I. Kid).

**Results:**

There was high NSSI frequency (39.9%–71% of them were girls) in our sample. NSSI was significantly more frequent among those who showed threshold symptoms on SDQ than in the subthreshold group [H(3) = 53.293, p <.001]. In the NSSI frequency, there was also a significant difference between ‘normal’ internet users and both ‘maladaptive’ and ‘pathological’ internet users [H(2) = 10.039, p <.05 p = .007]. According to the mediator models, the relationship between PIU and NSSI is not a direct association; it is mediated by all examined psychopathological factors (M.I.N.I. kid diagnoses) except for obsessive-compulsive disorder (OCD), alcohol abuse and dependence, and adjustment disorder.

**Conclusions:**

We found a high frequency of NSSI. According to our results, PIU in itself is not a risk factor for NSSI but might become a risk factor in the presence of comorbid psychiatric disorders. All of these findings draw the attention of clinicians to the importance of careful screening of comorbid disorders with PIU.

## Introduction

Due to the high prevalence of nonsuicidal self-injury (NSSI) in the past two decades, there is growing scientific interest in this phenomenon (1). It has become a proposed diagnosis in the 5th edition of the Diagnostic and Statistical Manual of Mental Disorders (2). The symptoms of NSSI in DSM-5 follow the main instructions of the definition of the ‘International Society for the Study of Self-injury’ (ISSS) made in 2007: NSSI is a deliberate self-injurious act with a nonsuicidal purpose, which is not socially sanctioned. It is important to distinguish it from drug overdoses, culturally sanctioned behaviours (*e.g.* piercings), and repetitive, stereotypical forms among people with developmental disorders (3). As a criterion for the proposed disorder, DSM-5 suggests: there should be 5 or more days in the past year when this kind of self-injurious acts happened. The nonadaptive ‘coping strategy’ nature of NSSI is also important: the individual who engages in NSSI must have the aim of reaching a better emotional state after the action (2).

Different risk factors, such as prior history of NSSI, cluster B personality, hopelessness, female gender, depression, prior suicidal thoughts/behaviour, exposure to peer NSSI, eating disorder, abuse, *etc.* are described (4). The associations between internalising psychopathology—when the symptoms are mainly presented towards the patient’s inner world, *e.g.* the symptoms of major depressive disorder (MDD) and anxiety disorders (5)—and NSSI are well studied (6, 7), and some studies in the past few years have examined the link between externalising psychopathology—when the symptoms are mainly presented towards the patient’s environment (5), *e.g.* the symptoms of conduct disorder (CD), oppositional defiant disorder (ODD), attention deficit hyperactivity disorder (ADHD)—and NSSI (8–10). To the best of our knowledge, NSSI still seems to be a multifactorial phenomenon, and whether it should be described as an individual diagnosis or a common symptom is still under discussion (11).

After a significant increase in the prevalence of NSSI at the beginning of the 21th century, prevalence rates have stabilised at high levels (12). This is the same period when internet spread all over the world (13). Content analysis studies repeatedly strengthen the cultural role of internet content in social media in the growth of NSSI prevalence in the general population *via* the influence of these web 2.0 based self-injury subcultural groups, where NSSI has become a culturally accepted phenomenon (14, 15). However, it is still unclear whether there is a specific association between pathological internet use (PIU) and NSSI or whether the broader use of internet is a risk factor for NSSI in itself.

PIU and ‘internet addiction’ are often used as synonyms. PIU has also been the focus of scientific research in the past two decades (16). In recent times, internet has become part of everyday life, especially among young people, although its effects on emotional and behavioural development are ambiguous (17). One type of ‘internet addiction’ is also a proposed diagnostic category in DSM-5: ‘Internet Gaming Disorder’ (2). Both this narrow category and the wider PIU are usually conceptualised as behavioural addiction disorders, with similar features to ‘Gambling Disorder’ (18). Definitions underline a preoccupation with internet use, withdrawal symptoms, tolerance, loss of control, loss of interest in old hobbies, excessive use of internet despite the knowledge of psychosocial problems, telling lies about the time spent on the internet, use of internet to reach a better emotional state, and the loss of anything important (job, friendship, *etc.*) in real life because of internet (2, 17, 18).

Although PIU and NSSI have similar risk factors, such as depression, anxiety, ADHD, higher rates of suicidal behaviour, impulse control problems, *etc.* (4, 18, 19), to the best of our knowledge there are only a few cross-sectional studies which have examined the association between PIU and self-injurious behaviours. In their recent systematic review, Marchant et al. (20) found only seven papers on this topic, with only two of them (21, 22) having investigated the connection between internet addiction and nonsuicidal self-injurious behaviours; they found a positive correlation between the two phenomena, while, interestingly, Aktepe et al. (22) found that adolescents with an internet addiction were less lonely and more satisfied with their lives. The rest of the papers explored a possible connection with potentially suicidal self-injurious behaviours (19, 22–26). All of the articles found a positive correlation between suicidal ideations and actions and internet addiction, as well as finding some common mediating factors between them, such as depression, anxiety, conduct problems, or ADHD (20).

After Marchant et al.’s (20) review period, additional four relevant papers were identified. Of these four articles, only Liu et al. (27) examined the possible association between PIU and NSSI: they found that internet addiction was related to an increased risk of self-harm with a hierarchical logistic regression analysis, after controlling for gender, family factors, exposure to suicidal thoughts in the real life, depression, alcohol/tobacco use, concurrent suicidality, and perceived social support. The other three articles (28–30) investigated the relationship between PIU and suicidal thoughts and actions; all three papers found a positive correlation.

To summarize the above, both internalising and externalising disorders are risk factors for NSSI. According to our hypothesis-1, in our sample NSSI is more frequent in all subgroups presenting clinical psychiatric symptoms (whether these symptoms are externalising, internalising, or mixed), compared to those who present only subthreshold symptoms or do not present symptoms. Earlier studies draw attention to the possible link between PIU and NSSI, but this association has been understudied. According to our hypothesis-2, there is a positive association between PIU and NSSI in our cross-sectional adolescent sample, but this connection is not a direct association; it is mediated by psychopathological factors from both internalising and externalising dimensions.

## Materials and Methods

The methodology of this study, including ethics, subjects, measures, has already been published by Balazs et al. (10) and Horvath et al. (31). We only describe below the core and some additional information on the methods. Our first previous study (10) contained a subsample of the current work; the previous analysis was performed only in the clinical part of the whole sample of this study. In the second study (31) a different statistical design was used, and the focus was on life events and its role in suicidal *versus* nonsuicidal self-injury.

### Ethics

The study was approved by The Ethical Committee of the Medical Research Council, Hungary (ETT-TUKEB), under study registration number 5750/2015/EKU. Both caregivers and participants who were older than 14 years old provided active written informed consent. They were informed both in written and oral forms about the study and had the opportunity to ask questions about the nature of the study. There was no refund for participating in the investigation.

### Sample

In line with the dimensional approach of psychiatric disorders, participants were recruited from both clinical and nonclinical settings. The clinical sample was enrolled in Vadaskert Child and Adolescent Psychiatric Hospital and Outpatient Clinic between 25.02.2015 and 09.05.2016. Inclusion criteria were age older than 13 years and being an inpatient in the acute adolescent patient ward of the hospital, where patients are hospitalized for an average of five days, then they are usually reintegrated in their everyday (*e.g.* school) environment. Exclusion criteria were serious psychiatric states (*e.g.* a severe psychotic episode) or mental retardation leading to a condition preventing the completion of self-administered questionnaires. Altogether, 33 patients were excluded. Of the remaining 224 patients, 22 refused to participate.

The nonclinical sample was collected from 22 classes of years 8–11 from high schools and vocational schools in Budapest, Hungary between 12.09.2015 and 28.04.2017. Of the 185 students whose parent/caregiver previously gave consent at the parent–teacher meetings, 10 students did not consent to participate; also, in three cases, the parent/child had their consent withdrawn, and participants were not available for data collection despite their consent in 11 cases (*e.g.* the student left the school before their data were collected, or was repeatedly absent on data collection appointments). Exclusion criteria in this group were the same as for inpatient participants; participants with prior or current psychiatric disorders and/or psychiatric treatment could be included in the nonclinical group.

### Measurements

Psychiatric symptoms and disorders according to DSM fourth edition (DSM-IV) (32) were evaluated by the modified version of Hungarian version of the modified Mini International Neuropsychiatric Interview Kid (M.I.N.I. Kid) 2.0 (33–36). The M.I.N.I. Kid was administered by trained MA psychology and medical students under strong continuous supervision by senior members of the study staff.

NSSI was evaluated by the Deliberate Self-Harm Inventory (DSHI) (37, 38), which is a self-administered questionnaire, with 17 questions on the method of self-injury being answered by a ‘yes’ or ‘no’. DSHI comprises facets on frequency, severity, and duration of self-injury as well.

PIU is evaluated by ‘Young Diagnostic Questionnaire for Internet Addiction’ (YDQ) (39), which is also a self-administered questionnaire. Eight items ask about problematic internet use behaviour in the past 6 months with a ‘yes’ or ‘no’ answer: common fantasies about internet, preoccupation with internet use, unsuccessful attempts to reduce internet use, emotional problems connected to reducing the use of internet, longer internet use than planned previously, loss of anything important (job, friendship, *etc.*) in real life because of internet, telling lies about time spent on internet, and use of internet to reach a better emotional state.

The ‘Strength and Difficulties Questionnaire’ (SDQ) (40) is used to evaluate psychopathology both in internalising and externalising dimensions. It is a widely used tool in the literature. The SDQ has an official Hungarian version, validated by Turi et al. (41). The SDQ is also a self-reported questionnaire with 25 items, which investigate five main domains. The two externalising scales are ‘hyperactivity/inattention’ and ‘conduct problems’, while the two internalising scales are ‘emotional symptoms’ and ‘peer relationship problems’; the 5th scale is ‘prosocial behaviour’. There are three versions of the SDQ, depending on who is completing the questionnaire: the child, the parents, or the teachers; in this study, we used the self-rated children version of SDQ, which was also validated without the parent version by Turi et al. (41).

### Study Groups

In line with the dimensional approach of psychiatric disorders, we assume that the mechanism between the assessed phenomena are comparable in both clinical and nonclinical populations, thus study groups were created based on participants’ internalising and externalising scores, regardless of the clinical/nonclinical setting of their recruitment. We have based our decision on the above described recruitment methods, prior evidence for the high prevalence of NSSI (42) and pathological internet use (16) in nonclinical populations, and prior evidence on full and subthreshold psychiatric disorders’ effect on everyday functioning and quality of life in both clinical and nonclinical settings (43).

According to appropriate statistical analysis, our subjects were divided into subgroups according to internalising and externalising disorders by their SDQ score. SDQ-internalising subgroup (1): sum of SDQ Emotional symptom subscale and Peer relationship problem subscale score equal to or greater than nine; SDQ-externalising subgroup (2): sum of SDQ Conduct problems subscale and Hyperactivity/inattention subscale score equal to or greater than nine; SDQ-internalising and externalising subgroup (3): both SDQ internalising and externalising subgroup scores equal to or greater than nine; SDQ subthreshold subgroup (4): both SDQ internalising and externalising subgroup scores less than nine. These cut-off points are based on the Hungarian standard of SDQ (Turi et al, 2013). Furthermore, we examined our subjects according to internet use: we divided our subjects into three subgroups: normal internet users (0–2 score on YDQ), maladaptive internet users (3–4 score on YDQ), and pathological internet users (more than five score on YDQ). These cut-off points are based on previous research (16, 39). Each YDQ question is very decisive between ‘addictive’ and ‘nonaddictive’ internet use behaviour, therefore only individuals with maximum one or two scores can fulfil normal internet user category. Consequently, maladaptive internet users and pathological internet users were considered as two pathological internet user groups.

### Statistics

IBM SPSS Statistics 22.0 was used to data analysis. Descriptive statistics are reported in the text. Mann–Whitney U tests were applied for continuous variables and chi-square tests were applied for categorical variables. The Kruska–Wallis test and Mann–Whitney U test were calculated to explore group differences across study subgroups. Spearman’s rank Correlation was applied to analyse the correlation between NSSI, internet use, and M.I.N.I diagnoses symptoms. Those M.I.N.I. Kid diagnoses that were comorbid with NSSI and had prevalence 7% or above 7% were included in mediator model analyses (**Table 1**). According to this, the following diagnoses were not included in the statistical analyses: Tourette’s syndrome, tic disorder, anorexia nervosa, bulimia nervosa, and autism spectrum disorder. To test the possible mediational effects of mental disorders on the relationship between internet use and NSSI, total, direct, and indirect effects were calculated using the mediation approach and SPSS PROCESS-Macro provided by Preacher and Hayes (44, 45). We conducted our analyses using dimensional approaches, that is why in the mediator model for mediator variables, we added the number of symptoms of the M.I.N.I. Kid diagnoses, and these continuous variables were used in the model. In case of affective disorders and anxiety disorders we made two bigger groups, in which we gathered the number of appropriate M.I.N.I Kid symptoms. In the mediator model, the independent variable was the score of YDQ, and the outcome variable was the number of self-harm-forms according to the Deliberate Self-Harm Inventory (DSHI) (10, 37). Gender and age were included as covariates. Related to NSSI outcome variable we used the sum of all 17 Deliberate Self-Harm Inventory (DSHI) items (10). Many individuals who self-injure use more than one method (37), so the outcome variable is calculated as a sum of YES answers of the types of self-harming behaviour occurring. Bootstrapping procedure (5,000 bootstrap sample) was used in the mediator model to show the significance because this method does not impose the assumption of normality of the sampling distribution (44).

**Table 1:** Prevalence of M.I.N.I Kid diagnoses.

M.I.N.I. Kid diagnoses	M.I.N.I. Kid diagnoses in total sample (N = 363)	M.I.N.I. Kid diagnoses—in total NSSI group (n = 145)	M.I.N.I. Kid diagnoses—in clinical NSSI group (n = 107)	M.I.N.I. Kid diagnoses—in nonclinical NSSI group (n = 38)
**Major depressive episode**	14.0%	28.5%	37.4%	2.7%
**Suicidality—lifetime**	46.8%	80.0%	88.8%	55.3%
**Suicidality—current**	31.4%	55.2%	68.2%	18.4%
**Dysthymic disorder**	9.1%	15.9%	17.8%	10.5%
**Manic episode—lifetime**	18.2%	30.3%	36.4%	13.2%
**Manic episode—current**	10.5%	19.3%	21.5%	13.2%
**Hypomanic episode—lifetime**	23.1%	25.5%	27.1%	21.1%
**Hypomanic episode—current**	10.7%	14.5%	15.0%	13.2%
**Panic disorder—lifetime**	15.9%	29.9%	36.4%	10.8%
**Panic disorder (limited symptoms)**	18.4%	26.4%	27.1%	24.3%
**Panic disorder—current**	12.0%	21.5%	27.1%	5.4%
**Agoraphobia—current**	23.1%	35.9%	43.0%	15.8%
**Agoraphobia with Panic disorder**	13.2%	24.1%	30.8%	5.3%
**Agoraphobia without Panic disorder**	9.9%	11.7%	12.1%	10.5%
**Separation anxiety disorder**	9.2%	16.7%	21.5%	2.7%
**Social anxiety disorder**	18.7%	32.6%	37.4%	18.9%
**Specific phobia**	10.9%	14.6%	15.9%	10.8%
**OCD**	20.1%	29.7%	37.4%	7.9%
**PTSD**	3.6%	6.9%	9.3%	0.0%
**Alcohol dependence**	9.8%	15.4%	15.5%	15.2%
**Alcohol abuse**	10.5%	15.2%	13.1%	21.1%
**Psychoactive substance dependence**	5.6%	9.8%	12.3%	2.7%
**Psychoactive substance abuse**	5.5%	9.0%	11.2%	2.6%
**Tourette**	1.1%	1.4%	1.9%	0.0%
**Vocal Tic**	0.3%	0.0%	0.0%	0.0%
**Tic transitions—current**	0.3%	0.0%	0.0%	0.0%
**ADHD combined type**	7.5%	12.5%	13.1%	10.8%
**ADHD inattention type**	9.5%	15.3%	19.6%	2.7%
**ADHD hyperactivity-impulsivity type**	3.9%	4.9%	3.7%	8.1%
**CD**	8.1%	13.9%	15.0%	10.8%
**ODD**	16.5%	28.5%	30.8%	21.6%
**Psychotic disorder—lifetime**	22.3%	38.9%	46.7%	16.2%
**Psychotic disorder—current**	8.9%	16.0%	17.8%	10.8%
**Affective disorder with psychotic symptoms**	5.3%	11.8%	15.9%	0.0%
**Anorexia nervosa**	5.2%	4.8%	6.5%	0.0%
**Bulimia nervosa**	1.9%	4.1%	4.7%	2.6%
**GAD**	2.0%	3.5%	4.7%	0.0%
**Adjustment disorder**	7.6%	11.5%	10.3%	14.7%
**Autism spectrum disorder**	5.0%	6.2%	8.4%	0.0%

The following grouped diagnoses were involved as mediator variables: a) affective disorders: major depressive episode, dysthymic disorder, hypo/manic episode; b) anxiety disorders: panic disorder, agoraphobia, separation anxiety disorder, social anxiety disorder, specific phobia, PTSD, GAD; c) OCD; d) ADHD; e) CD and ODD; f) alcohol abuse and dependence; g) psychoactive substance abuse and dependence; h) psychotic disorder; i) suicidality; and j) adjustment disorder.

The study is asserting statistical mediation with cross-sectional data.

## Results

### Subjects

The whole study population consisted of 363 adolescents, of whom 202 were included from clinical setting and 161 adolescents from nonclinical settings; mean age of participating adolescents (N = 363) was 15.12 years (SD = 1.31). About half of our population were girls (183 girls; 50.7%). In the total sample (N = 363), 145 (39.9%) adolescents had NSSI, with 42 (29%) boys and 103 (71%) girls. There were significantly more girls than boys among the adolescents with NSSI (χ2(1) = 41.071 p < 0.001 ϕ = 0.336). Prevalences of M.I.N.I Kid diagnoses are high among adolescents who reported NSSI, both in the clinical and in nonclinical sample. (**Table 1**).

### SDQ Symptoms and NSSI

Examining the differences across the four groups (SDQ-internalising, SDQ-externalising, SDQ-internalising and externalising, SDQ-subthreshold symptoms), we found a significant difference in the frequencies of NSSI (Kruskal–Wallis test: H(3) = 53.293, p < 0.001) (**Table 2**).

**Table 2:** Occurrence of NSSI by SDQ groups (how many people have NSSI by SDQ groups).

NSSI occurrence by adolescents	SDQ-internalizing	SDQ-externalizing	SDQ-internalizing and externalizing	SDQ-subthreshold symptoms
**NO**	34 (45.3%)	25 (47.2%)	16 (33.3%)	133 (77.8%)
**YES**	41 (54.7%)	28 (52.8%)	32 (66.7%)	38 (22.2%)
**Total**	75 (100%)	53 (100%)	48 (100%)	171 (100%)

(Related to SDQ we have data from 347 adolescents).

Bonferroni correction was applied to control for multiple comparison (p = 0.05/6 = 0.0083). Specifically, adolescents who were referred for SDQ internalising (1) reported significantly more NSSI compared with adolescents with SDQ subthreshold symptoms (4) (U = 4,045.000 z = −5.567 p < 0.001). No significant differences were found between adolescents with SDQ internalising (1) and SDQ externalising (2) (U = 1,946.000 z = −0.212 p = 0.832) and the SDQ internalising and externalising group (3) (U = 1,637.500 z = −0.876 p = 0.381). Adolescents who were referred for SDQ externalising group (2) reported significantly more NSSI compared with adolescents with SDQ subthreshold symptoms (4) (U = 2,945.000 z = −4.784 p<0.001). No significant differences were found between adolescents in the SDQ externalising group (2) and the SDQ internalising and externalising group (3) (U = 1,117.500 z = −1.093 p = 0.275). We found significantly more NSSI in the SDQ internalising and externalising group (3) compared with the SDQ subthreshold symptoms group (4) (U = 2,127.000 z = −6.170 p < 0.001).

We divided the whole sample into two groups (SDQ-threshold, SDQ subthreshold). Comparing the SDQ-threshold and SDQ-subthreshold group, adolescents who were referred for the SDQ threshold group reported significantly more NSSI than the SDQ subthreshold group (U = 9,117.000 z = −7.181 p < 0.001).

### Internet Use and NSSI Frequency

Examining the differences across the three internet use groups (normal, maladaptive, pathological), we found a significant difference in the frequencies of NSSI (Kruskal–Wallis test: H(2) = 10.039, p < 0.05 p = 0.007) (**Table 3**).

**Table 3:** Occurrence of NSSI by internet use groups (normal, maladaptive, pathological) (how many people have NSSI by internet use groups).

NSSI occurrence by adolescents	Normal Internet use	Maladaptive Internet use	Pathological Internet use
**NO**	160 (63.5%)	29 (50.9%)	8 (36.4%)
**YES**	92 (36.5%)	28 (49.1%)	14 (63.6%)
**Total**	252 (100%)	57 (100%)	22 (100%)

(Related to internet use we have data from 331 adolescents).

Bonferroni correction was applied to the control for multiple comparison (p = 0.05/3 = 0.017). Specifically, adolescents who were referred for maladaptive internet use reported significantly more NSSI compared with adolescents with normal internet use (U = 5,800.500 z = −2.586 p < 0.017 p = 0.010). We found significantly more NSSI in the pathological internet use group than in the normal internet use group (U = 2,020.000 z = −2.501 p < 0.017 p = 0.012). No significant differences were found between adolescents with maladaptive and pathological internet use (U = 625.500 z = −0.017 p > 0.017 p = 0.986).

### Tests of Mediation in the Association Between Internet Use and NSSI

According to our results, NSSI has significant correlation with internet use and all measured M.I.N.I Kid diagnoses symptoms. Internet use also has significant correlation with most M.I.N.I Kid diagnoses except for alcohol abuse and dependence and adjustment disorder (**Table 4**).

**Table 4:** Correlation between NSSI, internet use, and M.I.N.I Kid symptoms.

	NSSI		Internet use	
	Rs	p	Rs	p
**NSSI**			.203**	<.001
**Internet use**	.203**	<.001		
**Affective disorders**	.488**	<.001	.300**	<.001
**Anxiety disorders**	.446**	<.001	.220**	<.001
**OCD**	.322**	<.001	.124*	.023
**ADHD**	.308**	<.001	.285**	<.001
**Alcohol abuse and dependence**	.188**	<.001	.081	.153
**Psychoactive substance abuse and dependence**	.260**	<.001	.149**	.007
**CD and ODD**	.412**	<.001	.294**	<.001
**Psychotic disorder**	.383**	<.001	.255**	<.001
**Suicidality**	.508**	<.001	.267**	<.001
**Adjustment disorder**	.141*	.010	.030	.605

**p < .01.

*p < .05.

**Figure 1:**
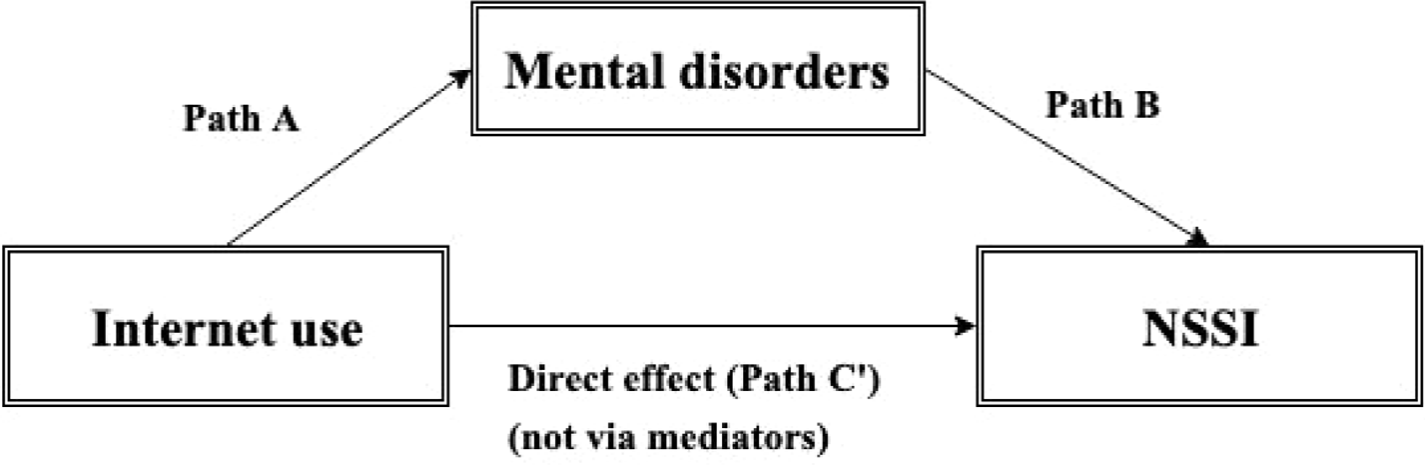
Mediation model. Path A, path B, the direct pathway/effect between internet use and NSSI (C′), and indirect pathway/effect *via* mediating factors (Path A * Path B). Path A: The effect of the symptoms of internet use on comorbid mental disorders. Path B: The effect of the comorbid mental disorders on prevalence of NSSI.

To test the possible mediational effects of M.I.N.I Kid diagnoses symptoms on the relationship between NSSI and internet use we calculated with mediation approach. Because of the results of Spearman’s rank correlation (**Table 4**), we decided that we use all M.I.N.I Kid diagnoses (affective disorders, anxiety disorders, OCD, ADHD, CD, and ODD, alcohol abuse and dependence, psychoactive substance abuse and dependence, psychotic disorder, suicidality, adjustment disorder) symptoms in mediator model as mediator variables. Before mediation analysis, we have checked the correlations between M.I.N.I Kid diagnoses (**Table 5**). According to the results there are correlations between them (**Table 5**) that is why we used M.I.N.I Kid diagnoses symptoms as mediator variables in ten different mediator models (**Figure 1**).

**Table 5:** Correlations of M.I.N.I Kid symptoms.

		Affective disorders	Anxiety disorders	OCD	ADHD	CD and ODD	Alcohol abuse and dependence	Psychoactive substance abuse and dependence	Suicidality	Psychotic disorder	Adjustment disorder
Affective disorders	Rs		.778^**^	.540**	.645**	.583**	.175**	.293**	.603**	.502**	.307**
	p		<.001	<.001	<.001	<.001	.001	<.001	<.001	<.001	<.001
Anxiety disorders	Rs	.778**		.608**	.579**	.582**	.126*	.263**	.519**	.498**	.425**
	p	<.001		<.001	<.001	<.001	.021	<.001	<.001	<.001	<.001
OCD	Rs	.540**	.608**		.411**	.344**	0.02	.136*	.420**	.323**	.288**
	p	<.001	<.001		<.001	<.001	.708	.010	<.001	<.001	<.001
ADHD	Rs	.645**	.579**	.411**		.710**	.157**	.201**	.365**	.401**	.255**
	p	<.001	<.001	<.001		<.001	.004	<.001	<.001	<.001	<.001
CD and ODD	Rs	.583**	.582**	.344**	.710**		.225**	.296**	.428**	.449**	.280**
	p	<.001	<.001	<.001	<.001		<.001	<.001	<.001	<.001	<.001
Alcohol abuse and dependence	Rs	.175**	.126*	0.02	.157**	.225**		.395**	.157**	.111*	.176**
	p	.001	.021	.707	.004	<.001		<.001	.004	.042	.002
Psychoactive substance abuse and dependence	Rs	.293**	.263**	.136*	.201**	.296**	.395**		.303**	.260**	.161**
	p	<.001	<.001	.010	<.001	<.001	<.001		<.001	<.001	.003
Suicidality	Rs	.603**	.519**	.420**	.365**	.428**	.157**	.303**		.392**	.252**
	p	<.001	<.001	<.001	<.001	<.001	.004	<.001		<.001	<.001
Psychotic disorder	Rs	.502**	.498**	.323**	.401**	.449**	.111*	.260**	.392**		.218**
	p	<.001	<.001	<.001	<.001	<.001	.042	<.001	<.001		<.001
Adjustment disorder	Rs	.307**	.425**	.288**	.255**	.280**	.176**	.161**	.252**	.218**	
	p	<.001	<.001	<.001	<.001	<.001	.002	.003	<.001	< 001	

**p < .01.

*p < .05.

Detailed results of mediations are presented in **Table 6**. Moreover **Table 7** summarizes the direct and indirect effects of mediation. Mediation results explain the relationship between internet use and NSSI. The pathway (C′)/direct effect shows direct relationship between internet use and NSSI. Mediation model shows that the independent variable (internet use) influences mediator variables (comorbid mental disorders) (Path A), which in turn influence the dependent variable (NSSI) (Path B). We apply Bonferroni correction in the mediation analyses to control for multiple comparison. (p = 0.05/10 = 0.005, p = 0.01/10 = 0.001). In the case of indirect effects, it is not possible to directly use Bonferroni correction while these are Bootstrapping 95% confidence intervals. Thus, we used Bonferroni correction in case of every path A and path B and we assume that if path A and path B are significant then the indirect effect is also significant. Although a Wald t-test could be conducted by calculating the proportion of the estimated effect and the bootstrapped standard error, if the sampling distribution of the statistic is not symmetric (and it is usually not symmetric in testing indirect effects), using t-statistic in a t-test would be invalid.

**Table 6:** Mediation of the effect of internet use on NSSI through mental disorders.

	Path A	Path B	Effect	SE	t	p	Bootstrapping 95% CI
**Mediator variable: Affective disorder** Total effect	1.335** p<.001	.140** p<.001	.183	.074	2.487	.013	
Direct effect			−.005	.068	−.067	.947	
Partial effect of control variables							
Gender			1.491**	.229	6.499	<.001	
Age			−.069	.086	−.802	.423	
Total indirect effect			.188*	.042			.119,.284
Model summary: R = .375, R2 = .140, F3, 326 = 17.759, p <.001							
**Mediator variable: Anxiety disorder** Total effect	1.905** p <.001	.064** p <.001	.183	.074	2.487	.013	
Direct effect			.061	.068	.889	.374	
Partial effect of control variables							
Gender			1.491**	.229	6.499	<.001	
Age			−.069	.086	−.802	.423	
Total indirect effect			.122*	.042			.050,.220
Model summary: R = .375, R2 = .140, F3, 326 = 17.759, p <.001							
**Mediator variable: OCD** Total effect	.155 p = .005	.432** p <.001	.183	.074	2.487	.013	
Direct effect			.116	.071	1.643	.101	
Partial effect of control variables							
Gender			1.491**	.229	6.499	<.001	
Age			−.069	.086	−.802	.423	
Total indirect effect			.067	.031			.018,.114
Model summary: R = .375, R2 = .140, F3, 326 = 17.759, p <.001							
**Mediator variable: ADHD** Total effect	.978** p <.001	.101** p <.001	.191	.074	2.597	.009	
Direct effect			.092	.075	1.229	.220	
Partial effect of control variables							
Gender			1.482**	.230	6.453	<.001	
Age			−.074	.086	−.858	.392	
Total indirect effect			.099*	.029			.053,.171
Model summary: R = .377, R2 = .142, F3, 324 = 17.867, p <.001							
**Mediator variable: CD and ODD** Total effect	.788** p <.001	.144** p <.001	.191	.074	2.597	.009	
Direct effect			.077	.074	1.048	.295	
Partial effect of control variables							
Gender			1.482**	.230	6.453	<.001	
Age			−.074	.086	−.858	.392	
Total indirect effect			.144*	.033			.059,.191
Model summary: R = .377, R2 = .142, F3, 324 = 17.867, p <.001							
**Mediator variable: Alcohol abuse and dependence** Total effect	.144 p = .036	.352** p <.001	.206	.078	2.640	.008	
Direct effect			.155	.075	2.074	.038	
Partial effect of control variables							
Gender			1.582**	.237	6.670	<.001	
Age			−.088	.088	−1.00	.318	
Total indirect effect			.051	.030			.007,.124
Model summary: R = .396, R2 = .157, F3, 306 = 18.968, p <.001							
**Mediator variable: Psychoactive substance abuse and dependence** Total effect	.201** p <.001	.405** p <.001	.188	.073	2.565	.011	
Direct effect			.107	.071	1.515	.131	
Partial effect of control variables							
Gender			1.467**	.230	6.374	<.001	
Age			−.070	.086	−.816	.415	
Total indirect effect			.081*	.033			.029,.163
Model summary: R = .374, R2 = .140, F3, 321 = 17.419, p <.001							
**Mediator variable: Psychotic disorder** Total effect	.325** p <.001	.356** p <.001	.191	.074	2.597	.009	
Direct effect			.075	.068	1.105	.270	
Partial effect of control variables							
Gender			1.482**	.230	6.453	<.001	
Age			−.074	.086	−.858	.392	
Total indirect effect			.116*	.037			.056,.204
Model summary: R = .377, R2 = .142, F3, 324 = 17.867, p <.001							
**Mediator variable** **Suicidality** Total effect	.217** p <.001	.686** p <.001	.183	.074	2.487	.013	
Direct effect			.034	.065	.524	.601	
Partial effect of control variables							
Gender			1.491**	.229	6.499	<.001	
Age			−.069	.086	−.802	.423	
Total indirect effect			.149*	.046			.072,.255
Model summary: R = .375, R2 = .140, F3, 326 = 17.759, p <.001							
**Mediator variable** **Adjustment disorder** Total effect	.118 p = .039	.269** p <.001	.196	.076	2.569	.010	
Direct effect			.165	.076	2.179	.030	
Partial effect of control variables							
Gender			1.555**	.242	6.436	<.001	
Age			−.069	.089	−.777	.438	
Total indirect effect			.032	.023			.001,.096
Model summary: R = .391, R2 = .153, F3, 297 = 17.877, p <.001							

**p <. 001.

*p <. 005.

Path A: The effect of the symptoms of internet use on comorbid mental disorders. Path B: The effect of the comorbid mental disorders on prevalence of NSSI.

Effect—unstandardized regression coefficients, SE—standard error of the unstandardized regression coefficients, Bootstrapping 95% CI—95% confidence interval, Number of bootstrap resample: 5,000.

Bonferroni correction was applied to the control for multiple comparison. (p = 0.05/10 = 0.005, p = 0.01/10 = 0.001).

This table shows the detailed statistical results of the 10 mediator models; in **Table 7** there is a short summary about the direct and indirect effect of each psychopathological group we examined.

**Table 7:** Direct and indirect effects of internet use on NSSI through mental disorders.

PIU—Mental disorders—NSSI mediation	Effect	SE	t	p	Bootstrapping 95% CI
**Mediator variable: Affective disorder**					
Direct effect	−.005	.068	−.067	.947	
Indirect effect	.188*	.042			.119,.284
**Mediator variable: Anxiety disorder**					
Direct effect	.061	.068	.889	.374	
Indirect effect	.122*	.042			.050,.220
**Mediator variable: OCD**					
Direct effect	.116	.071	1.643	.101	
Indirect effect	.067	.031			.018,.114
**Mediator variable: ADHD**					
Direct effect	.092	.075	1.229	.220	
Indirect effect	.099*	.029			.053,.171
**Mediator variable: CD and ODD**					
Direct effect	.077	.074	1.048	.295	
Indirect effect	.144*	.033			.059,.191
**Mediator variable: Alcohol abuse and dependence**					
Direct effect	.155	.075	2.074	.038	
Indirect effect	.051	.030			.007,.124
**Mediator variable: Psychoactive substance abuse and dependence**					
Direct effect	.107	.071	1.515	.131	
Indirect effect	.081*	.033			.029,.163
**Mediator variable: Psychotic disorder**					
Direct effect	.075	.068	1.105	.270	
Indirect effect	.116*	.037			.056,.204
**Mediator variable** **Suicidality**					
Direct effect	.034	.065	.524	.601	
Indirect effect	.149*	.046			.072,.255
**Mediator variable** **Adjustment disorder**					
Direct effect	.165	.076	2.179	.030	
Indirect effect	.032	.023			.001,.096

**p <. 001.

*p < .005.

Effect—unstandardized regression coefficients, SE—standard error of the unstandardized regression coefficients, Bootstrapping 95% CI—95% confidence interval, Number of bootstrap resample: 5,000.

Bonferroni correction was applied to the control for multiple comparison. (p = 0.05/10 = 0.005, p = 0.01/10 = 0.001).

According to the results (**Table 6**), except for OCD, alcohol abuse and dependence, adjustment disorder, all measured comorbid mental disorders were predicted by internet use. All measured comorbid mental disorders have significant effect on the appearance of NSSI (Path B). In the mediation model gender and age were used as covariates.

**Table 6** shows detailed information related to the total effect, direct effect and indirect effect (path A * path B) between internet use and NSSI. The total effect of internet use on NSSI can be expressed as the sum of the direct and indirect effects: C = C′ + A * B. Except for OCD, alcohol abuse and dependence, adjustment disorder, all other M.I.N.I diagnoses symptoms are significant indirect effect on the relationship of internet use and NSSI. In case of most mediator variables (affective disorders, anxiety disorders, ADHD, CD and ODD, psychoactive substance abuse and dependence, psychotic disorder, suicidality), results show there are only indirect effects (*via* mediators, Path A * Path B) between internet use and NSSI, and in case of all mediator variables direct effects (path C’ - direct effect - direct relationship between internet use and NSSI) are not significant. It means that the relationship between internet use (independent variable) and NSSI (dependent variable) was fully influenced (mediated) by comorbid mental disorders. According to our results affective disorders, anxiety disorders, ADHD and CD and ODD have the strongest relationship with internet use, and suicidality, OCD, psychoactive substance abuse have the strongest relationship with NSSI.

## Discussion

To the best of our knowledge, this is the first study investigating the previously found connection between PIU and NSSI (21, 22, 27), and whether this is a direct connection or is mediated by other cofactors, such as comorbid psychopathology among clinical and nonclinical adolescent samples.

Although according to our study design, prevalence rates cannot be the main focus, firstly, we would like to highlight the high frequency of NSSI in our sample; it was 39.9% (71% girls). In line with previous studies (46–49), this result in this population, which is collected from clinical and nonclinical settings, underlines the impact of NSSI among all adolescents. In our sample, the frequency of NSSI significantly differed between girls and boys. There is an argument in the literature about this difference: some authors highlight its importance (42, 50), while others deny it (51, 52).

A relatively high frequency of NSSI was found in ‘SDQ subthreshold group’. This finding supports the dimensional approach of our study because it shows that even in a group that can be considered ‘nonclinical’ based on the measurements, a serious mental health concern is very common. This is in line with those previous studies (43) underlining the role of subthreshold psychiatric symptoms in function-loss and in lower quality of life.

Our first hypothesis was supported. According to the comparison of psychopathology based on a screening instrument (SDQ-internalising, SDQ-externalising, SDQ-internalising and externalising, SDQ-subthreshold symptoms), there was no significant difference in NSSI frequency between the groups with pathology (SDQ-internalising, SDQ-externalising, and SDQ-internalising and externalising); however, all of them had a significantly higher NSSI frequency than the subthreshold group. These results support previous studies that underline the importance of externalising psychopathology in NSSI (8, 9) although earlier studies mainly focused on internalising disorders (4, 6, 53). According to our findings, NSSI seems to be a multifactorial phenomenon that is often comorbid with other psychiatric disorders, consistent with earlier studies (4, 6, 7, 9). This raises questions about the validity of an individual diagnosis as proposed by DSM-5 (2). So far, as NSSI is becoming a new diagnosis in next edition of DSM, comorbidity would become a rule rather than an exemption.

Our second hypothesis—that PIU and NSSI are connected to each other—was supported as well; we found this association when different internet user groups (‘Normal’, ‘Maladaptive’ and ‘Pathological’) were analysed considering their frequency of NSSI. The ‘Normal’ group had a significantly lower NSSI frequency than the ‘Maladaptive’ and ‘Pathological’ groups; however, there were no differences between the two pathological groups. These findings are similar to previous researches (21, 22, 27). According to the mediator model, PIU has a significant effect on all examined comorbid psychiatric categories, and all comorbid mental disorders predict the appearance of NSSI significantly, except for OCD, alcohol abuse and dependence, adjustment disorder. PIU in itself is not a risk factor of NSSI; it is only a risk factor when there are comorbid psychiatric disorders, especially affective disorders, anxiety disorders, ADHD, CD and ODD, psychoactive substance abuse and dependence, psychotic disorder, and suicidality. This underlines the importance of assessing internet use habits in a structured way for all patients in clinical settings. From a suicide prevention aspect, it is very important to identify these adolescents because there is a lot of evidence that NSSI is strongly connected to suicide (54).

There are a lot of aspects of NSSI, and its risk factors were not assessed by our current study. *E.g*, influence of pubertal development is very important in NSSI because mature emotion regulation skills are strong protective factors of NSSI (55), and this psychological dimension is developing in the age of adolescence. This could be a possible explanation why NSSI is the most frequent in adolescents and why its prevalence decreases after the mid twenties (47). In our previous studies we have already investigated the role of ADHD (10) and life events (31) and in our further studies we are planning to investigate other dimensions such as Axis II diagnoses or further possible protective factor for NSSI, *e.g.* coping mechanisms.

### Limitations

A limitation of our study was the cross-sectional design as casual relationships cannot be examined. Moreover, mediator variables may temporally occur between the predictors and criterion measures. In our mediation model we tested the mediation effect of mental disorders, but we did not assess all effects of other NSSI risk factors, such as hopelessness, abuse *etc.* It is a cross-sectional design, so causal relationship cannot be examined. Exactly the pathway is not known, so it might occur that dependent and independent variables are interchangeable because we do not exactly know which was first, pathological internet use and afterwards mental disorders, or the inverse. In addition, it could be that pathological internet use first emerged which predicts anxiety disorder, which may have an effect on depression, which can influence NSSI occurrence.

Many individuals who engage in NSSI use multiple methods to harm themselves (37). Instead of NSSI frequency in our current study we analysed the number of methods because according to Saldias et al.’s (56) study, the authors also analysed the number of methods of NSSI because it was felt to be reasonably reliable self-report indicators of NSSI compared to the frequency. According to the authors’ opinion, individuals with mental health problems are unable to accurately report the frequency due to the high number of NSSI episodes they had experienced. According to Black and Mildred’s (57) study, ‘retrospective information will make it more challenging to quantify exact frequencies, particularly when NSSI is a repetitive pattern (rather than an occasional act).’ In addition, Black and Mildred (57) suggest that engaging in high number of NSSI methods has also been linked to suicide attempts, so the number of NSSI methods is an important part of suicide risk. Given these studies and because of the high prevalence of mental disorders in our sample, we decided that analysis of NSSI methods is more reliable than NSSI frequency.

Another limitation of our study was the use of self-reported questionnaires (PIU, SDQ, DSHI), which can be biased: some participants may show their problems more intensely than they really feel them, while other participants can hide their symptoms.

Furthermore, possible selection bias might have occurred during recruitment, possibly affecting the involvement of youth with highest risk in the nonclinical group: *e.g.* students whose parents were repeatedly unavailable for consent, students who were repeatedly absent from school at the times of data collection, or adolescents who have already dropped out from formal education could not be involved in the study sample.

There is another possible limitation: a subsample of our current work was analysed before, which can decrease the statistical power of the current analysis (10, 31).

### Conclusions

n conclusion, we would like to highlight that the high frequency of NSSI, especially in the clinical group of adolescents with symptoms of PIU, calls the attention of the clinicians to the importance of routinely screening for NSSI in this population. Adolescents with symptoms of PIU and symptoms of comorbid psychiatric disorders need special focus. Early recognition and adequate treatment of the symptoms of PIU and comorbid conditions can be important in NSSI prevention among adolescents.

## Data Availability Statement

The datasets generated for this study are available on request to the corresponding author.

## Ethics Statement

The studies involving human participants were reviewed and approved by The Ethical Committee of the Medical Research Council, Hungary (ETT-TUKEB), under study registration number 5750/2015/EKU. Written informed consent to participate in this study was provided by the participants’ legal guardian/next of kin.

## Author Contributions

GM participated in the design of the study, performed the literature search, participated in the data collection and analyses, and drafted the manuscript. DG participated in the data collection and analyses, performed the statistical analyses and drafted the manuscript. LH participated in the design of the study, participated in data collection and analysis, and drafted the manuscript. DS participated in the data collection and analysis, and drafted the manuscript. JB was the principal investigator of the study, participated in the design of the study, coordinated the steps of the data analyses and drafted the manuscript. All authors contributed to the article and approved the submitted version.

## Funding

This work was supported by the Hungarian Scientific Research Fund (Hungarian abbreviation: OTKA, No: K-108336). This work was also supported by the ÚNKP-19-3 New National Excellence Program of the Ministry for Innovation and Technology. JB was supported by the János Bolyai Research Scholarship of the Hungarian Academy of Sciences. All funding sources had role in the design of the study and collection, analysis, and interpretation of data and in writing the manuscript.

## Conflict of Interest

The authors declare that the research was conducted in the absence of any commercial or financial relationships that could be construed as a potential conflict of interest.
